# Supplementary material for: The fate of hippocampal synapses depends on the sequence of plasticity-inducing events
Source: eLife. 2018 Oct 12;7:e39151. doi: 10.7554/eLife.39151 (PMC6205809; doi:10.7554/eLife.39151)
Supplement: Transparent reporting form [file elife-39151-transrepform.pdf]

eLIFE
1st Floor
24 Hills Road
Cambridge CB2 1JP, UK
P 01223 855340
W elifesciences.org
T @elife

eLife's transparent reporting form

We encourage authors to provide detailed information *within their submission* to facilitate the interpretation and replication of experiments. Authors can upload supporting documentation to indicate the use of appropriate reporting guidelines for health-related research (see EQUATOR Network), life science research (see the BioSharing Information Resource), or the ARRIVE guidelines for reporting work involving animal research. Where applicable, authors should refer to any relevant reporting standards documents in this form.

If you have any questions, please consult our Journal Policies and/or contact us: editorial@elifesciences.org.

Sample-size estimation

- You should state whether an appropriate sample size was computed when the study was being designed
- You should state the statistical method of sample size computation and any required assumptions
- If no explicit power analysis was used, you should describe how you decided what sample (replicate) size (number) to use

Please outline where this information can be found within the submission (e.g., sections or figure legends), or explain why this information doesn't apply to your submission:

As this is the first study using theta stimulation with single synapse read-out, it was not possible to estimate the efficacy of this induction protocol *a priori*. In the course of the study, we observed that the number of transfected CA3 neurons was critical for successful LTP induction. As described in the manuscript, we introduced a criterion for successful stimulation (dendritic calcium spikes) and performed experiments until we reached a sample size (~20) for statistically valid assessment of plasticity.
For the 7 day survival experiments, we started with a sample size similar to our previous study (Wiegert et al. 2013). As stated in the methods, several criteria had to be met during an experiment to make it into the final analysis (successful plasticity induction in the reporter neuron AND on the level of spine EPSCsTs, stable recording, no contamination of the culture). For the more complex experiment with multiple rounds of plasticity induction, we thus had to double the number of experiments to ~50 slice cultures per condition. Still, due to attrition according to the criteria stated above, the final *n* (at day 7) was not identical between treatment groups.

Replicates

- You should report how often each experiment was performed
- You should include a definition of biological versus technical replication
- The data obtained should be provided and sufficient information should be provided to indicate the number of independent biological and/or technical replicates
- If you encountered any outliers, you should describe how these were handled
- Criteria for exclusion/inclusion of data should be clearly stated
- High-throughput sequence data should be uploaded before submission, with a private link for reviewers provided (these are available from both GEO and ArrayExpress)

Please outline where this information can be found within the submission (e.g., sections or figure legends), or explain why this information doesn't apply to your submission:

The number of replicates (n) for each experiment is given in the figure legends (Fig. 1-3) or in the main text (sequential plasticity experiments). As we followed the fate of one stimulated spine per experiment, all replicates are biological replicates (different cultures from different animals). We did not encounter or exclude outliers. In experiments with multiple rounds of plasticity induction, we continued the protocol only when the first induction was successful (as described in the main text). Electrophysiological recordings were discarded when the series resistance changed more than 20% over the course of the experiment (as stated in the main text / methods section).

Statistical reporting

- Statistical analysis methods should be described and justified
- Raw data should be presented in figures whenever informative to do so (typically when N per group is less than 10)
- For each experiment, you should identify the statistical tests used, exact values of N, definitions of center, methods of multiple test correction, and dispersion and precision measures (e.g., mean, median, SD, SEM, confidence intervals; and, for the major substantive results, a measure of effect size (e.g., Pearson's r, Cohen's d)
- Report exact p-values wherever possible alongside the summary statistics and 95% confidence intervals. These should be reported for all key questions and not only when the p-value is less than 0.05.

Please outline where this information can be found within the submission (e.g., sections or figure legends), or explain why this information doesn't apply to your submission:

As stated in the methods section, data were tested for Gaussian distribution by D'Agostino & Pearson omnibus normality test. Normally distributed data were tested for significant differences with a two-tailed t-test (Fig. 3A) or one-way repeated-measures analysis of variance (ANOVA) followed by Sidak's multiple comparisons test (Fig. 3B, C). Data with non-normal distribution data were tested with the following nonparametric tests: Two-tailed Wilcoxon matched-pairs signed rank test (Fig. 2A, D, 3A), Friedman test followed by Dunn's multiple comparison test (Fig. 2C, S2A-C).

(For large datasets, or papers with a very large number of statistical tests, you may upload a single table file with tests, Ns, etc., with reference to sections in the manuscript.)

Group allocation

- Indicate how samples were allocated into experimental groups (in the case of clinical studies, please specify allocation to treatment method); if randomization was used, please also state if restricted randomization was applied
- Indicate if masking was used during group allocation, data collection and/or data analysis

Please outline where this information can be found within the submission (e.g., sections or figure legends), or explain why this information doesn't apply to your submission:

As all samples (organotypic hippocampal cultures) in our study were from male Wistar rats, there was no sample allocation procedure. Control experiments were mixed with plasticity induction experiments. The experimenter was not blind regarding the stimulation protocol applied. Data analysis was automatized with custom-written Matlab code.

eLife Sciences Publications, Ltd is a limited liability non-profit non-stock corporation incorporated in the State of Delaware, USA, with company number 5030732, and is registered in the UK with company number FC030576 and branch number BR015634 at the address 1st Floor, 24 Hills Road, Cambridge CB2 1JP | August 2014

Additional data files ("source data")

- We encourage you to upload relevant additional data files, such as numerical data that are represented as a graph in a figure, or as a summary table
- Where provided, these should be in the most useful format, and they can be uploaded as "Source data" files linked to a main figure or table
- Include model definition files including the full list of parameters used
- Include code used for data analysis (e.g., R, MatLab)
- Avoid stating that data files are "available upon request"

Please indicate the figures or tables for which source data files have been provided:

All individual data points are plotted in the figures. We will upload the numerical values of all plotted data as source data file (spread sheet).
